# Child Maltreatment, Mental Health Disorders, and Health Risk Behaviors in People With Diverse Gender Identities

**DOI:** 10.1177/08862605241270077

**Published:** 2024-08-17

**Authors:** Monica Madzoska, David Lawrence, Daryl J. Higgins, Divna M. Haslam, Ben Mathews, Eva Malacova, Michael P. Dunne, Holly E. Erskine, Rosana Pacella, Franziska Meinck, Hannah J. Thomas, James G. Scott

**Affiliations:** 1 Curtin University, Perth, WA, Australia; 2 Australian Catholic University, Melbourne, VIC, Australia; 3 Queensland University of Technology, Brisbane, QLD, Australia; 4 The University of Queensland, Brisbane, QLD, Australia; 5 Queensland Centre for Mental Health Research, Wacol, QLD, Australia; 6 John Hopkins University, Baltimore, MD, USA; 7 QIMR Berghofer, Medical Research Institute, Brisbane, QLD, Australia; 8 University of Greenwich, London, UK; 9 University of Edinburgh, Edinburgh, UK; 10 North-West University, Vanderbijlpark, South Africa; 11 University of the Witwatersrand, Johannesburg, South Africa; 12 Children’s Health Queensland, South Brisbane, QLD, Australia

**Keywords:** child abuse, sexual abuse, gender diversity, PTSD, mental health, neglect

## Abstract

This study examined rates of mental health disorders and health risk behaviors in people with diverse gender identities and associations with five types of child maltreatment. We used data from the Australian Child Maltreatment Study (ACMS), a nationally representative survey of Australian residents aged 16 years and more, which was designed to understand the experience of child maltreatment (physical abuse, sexual abuse, emotional abuse, neglect, exposure to domestic violence). Mental disorders—major depressive disorder, generalized anxiety disorder (GAD), alcohol use disorder, post-traumatic stress disorder (PTSD), and health risk behaviors—smoking, binge drinking, cannabis dependence, self-harm, and suicide attempt in the past 12 months were assessed. People with diverse gender identities who experienced child maltreatment were significantly more likely to have GAD (43.3%; 95% CI [30.3, 56.2]) than those who had experienced child maltreatment who were either cisgender men (13.8%; [12.0, 15.5]) or cisgender women (17.4%; [15.7, 19.2]). Similarly, higher prevalence was found for PTSD (21.3%; [11.1, 31.5]), self-harm (27.8%; [17.1, 38.5]) and suicide attempt (7.2%; [3.1, 11.3]) for people with diverse gender identities. Trauma-informed approaches, attuned to the high likelihood of any child maltreatment, and the co-occurrence of different kinds may benefit people with diverse gender identities experiencing GAD, PTSD, self-harm, suicidal behaviors, or other health risk behaviors.

## Introduction

Child maltreatment, including physical abuse, sexual abuse, emotional abuse, neglect, and exposure to domestic violence, is the most significant yet preventable public health risk factor for mental health disorders (McCrory et al., 2017; Teicher & Samson, 2016). The World Health Organization (WHO) reported that nearly three in four children between the ages of 2 and 4 years of age have endured physical or psychological abuse within the home (World Health Organization [WHO], 2022). Meta-analyses of self-report studies have generated global estimates of the prevalence of physical abuse (22.6%), sexual abuse (12.7%), and emotional abuse (36.3%; Stoltenborgh et al., 2011, 2012, 2013) demonstrating that child maltreatment in various forms is a significant issue across the globe. Similar prevalence estimates of child maltreatment and the associated mental health conditions have been reported across developed countries including Australia, North America, and Europe with similar patterns of maltreatment found across developed countries (Stoltenborgh et al., 2013). Several studies have demonstrated that while maltreatment directly impacts the mental health of children, it also subsequently increases the risk of poor mental health and physical health in later adulthood (Brühl et al., 2019; Molnar et al., 2001; Ramos et al., 2022). Many survivors of child abuse report clinically significant mental health symptoms (Lueger-Schuster et al., 2014) have academic difficulties (Ochoa & Constantin, 2023), have a high risk of eating disorders (Caslini et al., 2016), and are more likely to have mental health conditions such as post-traumatic stress disorder (PTSD), obsessive–compulsive disorder, anxiety disorders, and somatoform disorders (Liveri et al., 2023).

Despite the well-established association between child maltreatment and adult health harms, there are limited data on co-occurrence of all five forms of child maltreatment experienced from infancy through to age 18 years and the extent to which each maltreatment type is associated with subsequent mental health disorders (Haslam et al., 2023). Although child maltreatment stands as a pervasive issue in the general population (Feng et al., 2023; Negriff, 2020), relatively few studies have examined its impact on individuals identifying with a diverse gender identity, that is, a person whose gender identity differs from their sex assigned at birth (Poteat et al., 2023). In the United States, approximately three quarters of gender diverse individuals have experienced emotional abuse, nearly one in two physical abuse, and one in five sexual abuse (Thoma et al., 2021). Additional studies have shown that more than one in two gender diverse youth report instances of childhood sexual abuse and emotional abuse (Schnarrs et al., 2019; Streed et al., 2023). In addition to the limitations of previous research on gender diverse health, there is a need to determine how multi-type maltreatment (MTM), that being two or more forms of maltreatment being experienced, impacts mental health, specifically that of gender diverse youth. There has been a substantial increase in the proportion of young people identifying as gender diverse across developed countries, and the mental health and wellbeing of gender diverse young people has become a major issue for health service reform in several countries (Coleman et al., 2022 ).

### Adverse Childhood Experiences and Maltreatment Among Gender Diverse Populations

A recent Australian study of transgender youth has highlighted the disproportionately high rates of adverse childhood experiences (ACEs) in this group (Strauss et al., 2020). ACEs encompass aspects of familial adversities experienced in childhood such as parental death, parental incarceration, and parental substance use, as well as experiences of sexual abuse, emotional abuse, physical abuse, and physical neglect (Felitti et al., 1998). A nationwide online survey in the United States highlighted that around three quarters of individuals with diverse gender identities have experienced emotional abuse in childhood, around two in five have been subjected to physical abuse, and one in five to sexual abuse (Thoma et al., 2021). Other studies have reported elevated rates of sexual abuse, unwanted sexual contact, and emotional abuse toward this group, all of which were associated with minority stressors such as homophobia, stigma, and familial rejection (Bailey et al., 2023; Sizemore et al., 2022). Familial rejection and stigmatized attitudes toward gender diverse individuals have been found to be associated with intrafamilial and extrafamilial sexual and physical abuse; and they can lead to poorer mental health in children and adults with diverse gender identities (Andersen & Blosnich, 2013).

Subtypes of child maltreatment among diverse gender individuals have often been studied through the lens of ACEs. While household dysfunction and familial adversities negatively impact mental health, some researchers have suggested that the child maltreatment items within the broader ACEs framework have stronger effects on negative life outcomes than other childhood adversities (Atzl et al., 2019; Negriff, 2020), raising the question of whether a more thorough examination of the specific impacts of child maltreatment is warranted. While household dysfunction and child maltreatment often co-occur, previous research has often studied the increased risk of adversities overall rather than focusing specifically on child maltreatment, which is the key focus of the current study. Studies have reported the experience of child maltreatment and the impacts among gender diverse populations; however, the data are limited to gender diverse people, and have not assessed comparative data with cisgender individuals (Thoma et al., 2021; Tran et al., 2023). It is therefore not known to what extent gender diverse individuals are at increased risk of childhood adversities and have poorer mental health as a result, while comparing with the broader population (Strauss et al., 2017).

### Mental Health Problems in Gender Diverse People

A recent systematic review reported that transgender and other gender diverse children and adolescents are at a greater risk of developing mental health disorders than those who identify as cisgender, partly due to increased peer victimization and abuse related to their gender identity (Tran et al., 2023). Consistent with this, studies have reported that gender diverse people are around three times more likely than their cisgender counterparts to be diagnosed with a mental illness (Bailey et al., 2023; Hill et al., 2021; Reisner et al., 2015). In particular, PTSD has been reported to be five times more likely in gender diverse youth (Nuttbrock et al., 2014); suicidal ideation has also far been found to be more common among gender diverse individuals, with 23.6% of transgender women and 26.7% of nonbinary assigned male at birth reporting suicidal ideation (compared with cisgender women; 6.6%, cisgender men: 6.1%) (Kirakosian et al., 2023). The Trans Pathways Study showed that gender diverse youth also have high rates of health risk behaviors and chronic health conditions (Strauss et al., 2017). Prior literature has pointed out that health risk behaviors (i.e., alcohol and substance use, sexual risky behaviors) are often linked to the sexual or emotional abuse endured as a child (Becerra-Culqui, 2018; Craig et al., 2020; Sherman et al., 2022).

### Child Maltreatment and Associated Mental Health Problems in Gender Diverse Youth

It remains an open question as to what extent child maltreatment contributes to long-term psychopathology in gender diverse youth and older adults, and whether these associations differ from cisgender populations (Higgins et al., 2024). Youth who express themselves outside of the “norms” of societal expectations of gender make them more susceptible to being a target of abuse (Higgins et al., 2024). The higher rates and risk of abuse toward gender diverse individuals may partially explain the excessive mental health problems reported among this group; however, this remains unclear in available literature. Research into the prevalence of child maltreatment and associated health outcomes in people with diverse gender identities is required to understand the increased morbidity experienced in this population. This will inform preventative interventions and service provision.

### Child Maltreatment and Associated Outcomes Among Gender Diverse People in Australia

The Australian Child Maltreatment (ACMS) is the first nationally representative study to assess retrospectively all five types of child maltreatment over the entire span of childhood through to age 18 years (Mathews et al., 2020). The study found that among children who have been maltreated, it was most common to experience three to five forms of maltreatment (23.3%; Higgins et al., 2023). Approximately 48% of participants who had experienced child maltreatment met diagnostic criteria for a mental disorder, in comparison to 21.6% of those who did not experience maltreatment (Scott et al., 2023). However, this comparative data has not yet been analyzed among the gender diverse sample using the ACMS data. Reporting on data from the ACMS, Higgins et al. (2024) found an increased risk of child maltreatment in those with diverse identities. In particular, participants with diverse gender identities were more commonly exposed to child maltreatment compared to the full sample (81.5% vs. 62.0%). This was found for all five types: physical abuse (49.9% vs. 32.0%), sexual abuse (51.9% vs. 28.5%), emotional abuse (58.3% vs. 30.9%), neglect (26.4% vs. 8.9%), and exposure to domestic violence (58.2% vs. 39.6%; Mathews, Pacella et al., 2023). While it was estimated that females are more likely to experience child maltreatment than males, the associations between maltreatment and mental disorders were similar in strength in both males and females (Scott et al., 2023). This study builds on the existing nationally representative reports of child maltreatment, examining experiences among the gender diverse group of participants. This analysis goes beyond the reporting of prevalence of child maltreatment among gender diverse people, to further investigate the probable long-term impacts of child maltreatment and whether the harms experienced by people with diverse gender identities may contribute to their comparatively high risk of mental health problems, while also comparing prevalence with cisgender individuals.

## Methods

### Study Design and Participants

This study used ACMS data, a nationally representative sample of 8,503 Australians aged 16 years and over about their experiences of child maltreatment, and diagnostically measured mental disorders and assessed self-reported health risk behaviors. The study used a cross-sectional retrospective interview administered via random digit dialing and interviewed using computer-assisted telephone interviewing (Haslam et al., 2023). The full methodology of the survey has been previously published (Haslam et al., 2023). The sample was stratified by age and included an over-sample of young people aged 16 to 24 years (*n* = 3,500), as well as 1,000 participants in each of five successive 10-year age groups from 25 to 34 years up to those aged 65 and above. The Queensland University of Technology Human Research Ethics Committee approved this study (1900000477). All participants provided informed consent.

### Measures

#### Child Maltreatment

Child maltreatment was assessed using the Juvenile Victimization Questionnaire—R2: Adapted Version (Australian Child Maltreatment Study; Mathews, Meinck et al., 2023). The 16 screener items measured all 5 maltreatment types: physical abuse (2 items), sexual abuse (4 items), emotional abuse (3 items), neglect (3 items), and exposure to domestic violence (4 items). All screeners had a dichotomous “yes/no” response option, as well as “don’t know” and “prefer not to say” options (Haslam et al., 2023). Multitype maltreatment (MTM) was accounted for by experience of one type of child maltreatment, two types of maltreatment, or three or more types of maltreatment (referred to, for the purposes of this article, as severe MTM).

#### Gender Identity

We developed an item to measure gender identities after consultation with experts, closely aligned to the new standards outlined by the Australian Bureau of Statistics (2021), which is responsible for the 5-yearly national census, and a range of other key government surveys and national data collections. Participants were asked “How would you describe your gender?” Interviewers coded responses as man/cisgender, woman/cisgender, trans woman, trans man, trans femme, transmasculine, gender queer, gender diverse, gender fluid, nonbinary, sistergirl, brotherboy, agender, or “I prefer not to have a label.” Biological sex or sex at birth was not recorded. Responses of “man/cisgender” and “woman/cisgender” were categorized as “cisgender” (Supplemental Table 1). We use “diverse gender identities” and/or “gender diverse individuals” to include all gender identities other than man/cisgender and woman/cisgender, including trans and nonbinary people due to the small number of people who identified with diverse gender identities (McIntyre, 2018). As such, the analyses include three mutually exclusive categories of gender: cisgender men, cisgender women, and individuals with diverse gender identities.

#### Mental Disorders

The Mini-International Neuropsychiatric Interview Version 7.0.2 (MINI) (12) was administered to diagnose mental disorders. The MINI is a brief, structured diagnostic interview instrument designed to be administered by trained lay interviewers (Sheehan et al., 1998). Four mental disorders were assessed according to diagnostic criteria established in the Diagnostic and Statistical Manual for Mental Disorders (DSM-5): generalized anxiety disorder (GAD; current); PTSD (current); alcohol use disorder (AUD; current); and major depressive disorder (MDD; lifetime).

#### Health Risk Behaviors

Tobacco use and alcohol use were measured using items adapted from the 2007 Australian National Survey of Mental Health and Wellbeing (Slade, 2009). This included cigarette smoking in the past 12 months and binge drinking (six or more drinks for males or five or more drinks for females in a single session at least weekly over the past 12 months), based on gender assigned at birth. This survey question, taken from the Australian National Survey of Mental Health and Wellbeing was based on the Australian National Health and Medical Research Council’s guidelines on alcohol consumption. These guidelines have since been revised to advise no more than four standard drinks in a single session for cisgender males, females, and people with diverse gender identities alike. Cannabis dependence was measured using the 2006 Cannabis Severity of Dependence Scale, with dependence indicated by a score of 3 or more (Martin et al., 2006). Suicide attempts and self-harm in the past 12 months were assessed using items adapted from the National Adolescent Mental Health Surveys (Erskine et al., 2023). For the purpose of these analyses, this included the following questions related to self-harm “In the past 12 months have you deliberately harmed or injured yourself, without intending to end your own life?” and suicide attempts “Have you attempted suicide?”

### Statistical Analysis

Survey data were weighted, taking into account selection and response probabilities to match the population distribution of adults aged 16 years and more in Australia (Haslam et al., 2023). The weighted prevalence of each mental disorder and health risk behavior was calculated separately for the three categories of gender: those identifying as men, women, or individuals with a diverse gender identity. These were then further disaggregated by experience of child maltreatment.

We fitted separate logistic regression models to evaluate the association of each mental disorder and health risk behavior with experience of child maltreatment, by gender and age group. Two sets of models were run. The first model adjusted for gender, age group, and experience of child maltreatment. The second model also adjusted for financial hardship during childhood, current financial strain, and socioeconomic disadvantage (based on postcode of residence and quintiles of the Index of Relative Socio-Economic Disadvantage)—for which there were no significant differences. All analyses were conducted using SAS Version 9.4 (SAS Institute Inc., Cary, NC, USA). Logistic regression models used survey weights and adjusted for the complex sample design using the Surveylogistic procedure; 95% confidence intervals were calculated for all estimates using the Taylor series method of expansion.

## Results

The ACMS sample included 8,503 participants: 4,195 cisgender males (49.3%), 4,182 cisgender females (49.2%), and 126 people who identified with a diverse gender (1.5% of the sample, unweighted; Table 1). Within the youth sample aged 16 to 24 years, the proportion of individuals with diverse gender identities was higher than other age strata (2.3%, *n* = 90).

**Table 1.** Sample Characteristics.

Characteristics	Cisgender Men	Cisgender Women	Diverse Gender Identities
	Sample Size		
Age group			
16–24 years	1,748	1,662	90
25–44 years	992	986	22
45 years or more	1,455	1,534	14
Total 16 years or more	4,195	4,182	126
Prevalence of mental disorder			
Post-traumatic stress disorder (current)	4.4 [3.6, 5.3]	5.9 [5.1, 6.8]	**17.5** [8.9, 26.1]
Generalized anxiety disorder (current)	9.8 [8.6, 10.9]	13.0 [11.7, 14.2]	**38.0** [26.3, 49.6]
Major depressive disorder (lifetime)	15.4 [14.0, 16.7]	21.2 [19.7, 22.8]	16.9 [8.7, 25.1]
Alcohol use disorder (current)	24.1 [22.5, 25.8]	15.0 [13.7, 16.3]	19.9 [10.7, 29.1]
Any mental disorder	38.0 [36.2, 39.9]	37.7 [35.9, 39.5]	**52.7** [40.0, 65.4]
Prevalence of risk behaviors			
Current smoker	19.5 [17.9, 21.1]	15.1 [13.7, 16.6]	25.6 [14.3, 36.8]
Binge drinking	15.6 [14.2, 17.0]	6.7 [5.7, 7.7]	11.6 [3.2, 19.9]
Cannabis dependence	3.3 [2.6, 4.0]	1.7 [1.2, 2.2]	7.3 [0.6, 14.0]
Self-harm in past 12 months	2.2 [1.7, 2.7]	3.8 [3.1, 4.4]	**23.7** [14.5, 32.9]
Suicide attempt in past 12 months	0.9 [0.6, 1.3]	1.1 [0.8, 1.4]	**5.9** [2.6, 9.2]

*Note*. Where prevalence among people with diverse genders identities is significantly different from both men and women this is indicated in bold.

### Mental Disorders Among Gender Diverse Individuals

The overall prevalence of any mental disorder was significantly higher in individuals with diverse gender identities compared with cisgender men and cisgender women (Table 1). This was true for PTSD, as well as for GAD with over one-third of people with diverse gender identities meeting diagnostic criteria. The prevalence of MDD and AUD was not significantly different among people with diverse gender identities and cisgender men and women (Table 1).

### Association Between Child Maltreatment and Mental Disorders in Individuals With Diverse Gender Identities

**Figure 1.**
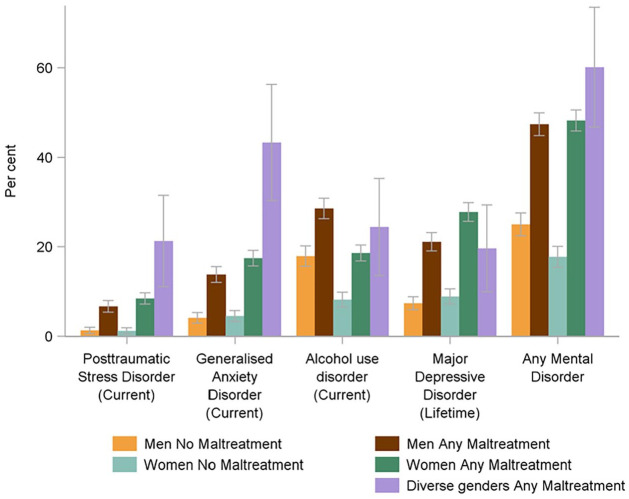
Prevalence of mental disorders, by gender identity and experience of any child maltreatment.

Among those who had experienced child maltreatment, prevalence of mental disorders was highest in people with diverse gender identities (Figure 1). PTSD and GAD were significantly more common in maltreated individuals with diverse gender identities. More than one in five maltreated individuals with diverse gender identities met criteria for PTSD, compared with less than one in ten cisgender men and cisgender women who had experienced child maltreatment (Figure 1). Almost half of maltreated individuals with diverse gender identities met criteria for GAD, compared with less than one in five cisgender men and cisgender women who experienced child maltreatment (Figure 1). The prevalence of any mental disorder was highest among gender diverse individuals who experienced sexual abuse and emotional abuse. The prevalence of GAD and PTSD was mostly associated with emotional abuse and neglect.

**Figure 2.**
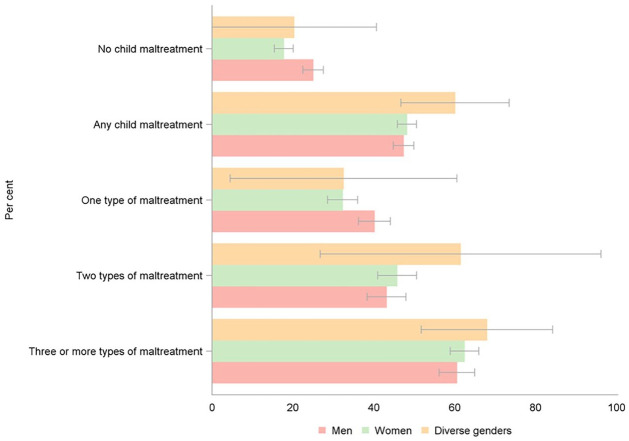
Prevalence of any mental disorders, by gender identity and number of types of maltreatment experienced.

The same pattern of mental disorders was evident when MTM was examined. Confidence intervals were wide due to small cell sizes and most differences did not reach statistical significance (Figure 2). Again, a pattern of comparatively larger differences between gender diverse and cisgender men and women was evident for PTSD and GAD (Supplemental Table 2).

### Prevalence of Health Risk Behaviors Among Those With Diverse Gender Identities

While health risk behaviors were common in people with diverse gender identities, not all behaviors were significantly more prevalent than cisgender respondents. Substance use did not differ significantly between individuals with diverse gender identities and cisgender individuals. About 1 in 14 gender diverse individuals had cannabis dependence, compared to less than 1 in 30 (Table 1). By contrast, for both self-harm and suicide attempts in the past 12 months, people with diverse gender identities had significantly higher rates than cisgender men and cisgender women. Almost one in four people with diverse gender identities had self-harmed in the previous 12 months compared with less than 1 in 20 cisgender men and cisgender women. People with diverse gender identities also had substantially higher rates of attempted suicide in the previous 12 months, compared with cisgender men and women. Although people with diverse gender identities had a younger age distribution and self-harm and suicidal behaviors were more common at younger ages, these stark gender differences were also observed when restricted to people aged 16 to 24 years only (Supplemental Table 3).

### Association Between Child Maltreatment and Health Risk Behaviors in Individuals With Diverse Gender Identities

**Figure 3.**
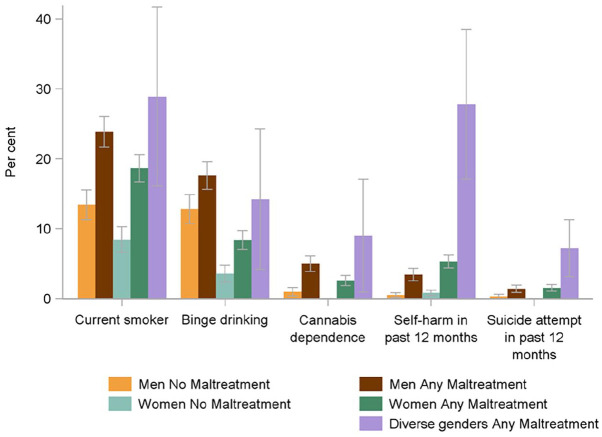
Prevalence of health risk behaviors, by gender identity and experience of any child maltreatment.

Among those who experienced child maltreatment, several health risk behaviors were more prevalent in individuals with diverse gender identities, including cannabis dependence, self-harming, and suicide attempts (Figure 3). Again, these patterns were similar among the youth cohort, suggesting that the higher prevalence of these risk behaviors cannot be explained by the younger age profile of people with diverse gender identities alone.

Prevalence of health risk behaviors in people with diverse gender identities who experienced child maltreatment varied by maltreatment type, and number of types of maltreatment experienced. Health risk behaviors were more commonly reported among people with diverse gender identities with a history of sexual abuse, physical abuse, and neglect. For example, cannabis dependence was most frequently associated with physical abuse and sexual abuse. Health risk behaviors were more common in individuals with diverse gender identities who experienced three or more types of child maltreatment in comparison to people with diverse gender identities who experienced fewer types of maltreatment. For instance, among gender diverse individuals who experienced three or more types of child maltreatment, smoking, binge drinking, cannabis dependence, self-harm, and suicide attempt were all elevated (Supplemental Table 5).

Table 2 presents the results of the logistic regression analyses. Separate models were fitted for each mental disorder and health risk behavior. After adjusting for age, people with diverse gender identities who experienced child maltreatment were significantly more likely to have PTSD, GAD, MDD, and any mental disorder compared to cisgender men and cisgender women who had not experienced child maltreatment. After further adjusting for financial strain, financial hardship in childhood, and socioeconomic status, odds ratios remained significantly elevated for PTSD, GAD, and any mental disorder. Odds of AUD did not significantly vary by gender or experience of child maltreatment. Odds of MDD were significantly greater for those who had experienced child maltreatment, but similarly elevated rates were observed for both cisgender individuals and individuals with diverse gender identities. After adjusting for age, financial strain, financial hardship in childhood, and socioeconomic status, health risk behaviors, specifically, cannabis dependence, self-harm, and suicide attempt were substantially more likely in individuals with diverse gender identities with a history of child maltreatment compared to cisgender individuals with no history of child maltreatment.

**Table 2.** Likelihood of Mental Disorders and Health Risk Behaviors, by Gender Identity and Experience of Child Maltreatment.

	Cisgender Men, No Maltreatment	Cisgender Women, No Maltreatment	Cisgender Men, Any Maltreatment	Cisgender Women, Any Maltreatment	Diverse Gender Identities, Any Maltreatment
Adjusting for age (odds ratio [95% confidence interval])					
Post-traumatic stress disorder	1.0 (ref)	0.9 [0.4, 2.1]	5.4 [3.0, 9.6]	7.0 [4.0, 12.3]	17.6 [7.8, 39.6]
Generalized anxiety disorder	1.0	1.2 [0.8, 1.7]	3.7 [2.6, 5.2]	5.0 [3.6, 6.9]	14.4 [8.0, 25.8]
Alcohol use disorder	1.0	0.4 [0.3, 0.6]	1.8 [1.5, 2.2]	1.0 [0.8, 1.3]	1.2 [0.7, 2.3]
Major depressive disorder	1.0	1.3 [0.9, 1.7]	3.3 [2.6, 4.2]	4.8 [3.8, 6.1]	2.7 [1.4, 5.2]
Any mental disorder	1.0	0.7 [0.5, 0.8]	2.7 [2.3, 3.2]	2.8 [2.4, 3.4]	3.7 [2.1, 6.6]
Current smoking	1.0	0.6 [0.4, 0.8]	2.0 [1.6, 2.5]	1.5 [1.2, 1.8]	2.6 [1.3, 4.9]
Binge drinking	1.0	0.2 [0.2, 0.4]	1.4 [1.2, 1.8]	0.6 [0.5, 0.8]	1.2 [0.5, 2.9]
Cannabis dependence	1.0	0.1 [0.0, 0.4]	5.4 [2.7, 10.6]	2.7 [1.4, 5.6]	7.4 [2.2, 25.2]
Self-harm in past 12 months	1.0	1.8 [0.8, 4.1]	7.3 [3.6, 14.8]	12.0 [6.0, 23.8]	52.4 [22, 123]
Suicide attempt in past 12 months	1.0	1.0 [0.2, 5.4]	4.8 [1.6, 14.0]	5.3 [1.9, 15.4]	16.0 [5.2, 49.1]
Adjusting for age, financial hardship in childhood, current financial strain, and socioeconomic status					
Post-traumatic stress disorder	1.0	0.9 [0.4, 1.9]	4.1 [2.3, 7.4]	4.7 [2.7, 8.5]	9.5 [3.7, 24.2]
Generalized anxiety disorder	1.0	1.1 [0.7, 1.7]	3.0 [2.2, 4.3]	3.8 [2.7, 5.3]	8.6 [4.6, 16.2]
Alcohol use disorder	1.0	0.4 [0.3, 0.5]	1.7 [1.4, 2.0]	0.9 [0.7, 1.1]	1.0 [0.5, 1.9]
Major depressive disorder	1.0	1.3 [0.9, 1.7]	3.0 [2.4, 3.9]	4.4 [3.4, 5.6]	2.5 [1.3, 4.8]
Any mental disorder	1.0	0.7 [0.5, 0.8]	2.4 [2.0, 2.8]	2.4 [2.0, 2.8]	2.8 [1.6, 5.1]
Current smoking	1.0	0.6 [0.4, 0.8]	1.8 [1.4, 2.2]	1.2 [1.0, 1.5]	1.7 [0.9, 3.2]
Binge drinking	1.0	0.2 [0.2, 0.4]	1.3 [1.0, 1.6]	0.5 [0.4, 0.7]	1.0 [0.4, 2.4]
Cannabis dependence	1.0	0.1 [0.0, 0.4]	4.5 [2.3, 9.1]	2.1 [1.0, 4.4]	4.3 [1.2, 15.4]
Self-harm in past 12 months	1.0	1.7 [0.8, 3.9]	6.4 [3.2, 13.1]	10.0 [5.0, 20.0]	40.7 [16.6, 99.6]
Suicide attempt in past 12 months	1.0	0.9 [0.2, 4.9]	3.9 [1.3, 11.5]	3.8 [1.3, 11.0]	8.7 [2.7, 27.4]

## Discussion

This article extended previous work examining the prevalence of gender diversity and the associations with five types of child maltreatment (Higgins et al., 2024). The results revealed that for gender diverse people, the experience of child maltreatment is more significantly associated with mental health disorders and health risk behaviors when compared to cisgender men and women. The rate at which gender diverse people are more likely to report symptoms of PTSD, GAD, self-harm, attempted suicide, and cannabis dependence may partially result from the increased rates of intrafamilial and extrafamilial maltreatment perpetrated toward gender diverse youth. ACMS data provide a rare opportunity to examine associations between child maltreatment, mental disorders, and health risk behaviors among people with diverse gender identities in a nationally representative sample. Prevalence of mental disorders was especially high among gender diverse youth, with 75.1% current or lifetime mental disorders diagnoses, compared to 54.5% of cisgender men and 63.8% of cisgender women. The high prevalence of child maltreatment and the strong relationship between experience of maltreatment and mental disorder suggest that child maltreatment may be an underlying factor in a high proportion of mental disorder cases. As the prevalence of mental disorders is high across the developed world (Kessler & Üstün, 2008), child maltreatment may contribute to a high proportion of mental disorder cases worldwide.

### Gender Diversity and Child Maltreatment Exposure

The data from the ACMS show that a remarkably high proportion (81%) of diverse gender people have a history of child maltreatment. There are few groups in society where four in every five people have been abused or neglected as children. The finding that individuals with diverse gender identities more commonly experience all five types of child maltreatment is unfortunately consistent with several recent studies (Kirakosian et al., 2023; Sherman et al., 2022; Tran et al., 2023). Among both, people with diverse gender identities and cisgender individuals, those who experienced sexual abuse and emotional abuse were substantially more likely to have poorer mental health in their adulthood. However, the strength of the association between mental disorders and maltreatment appears somewhat greater among those who have a diverse gender identity. Using the ACMS data, Scott et al. (2023) concluded that while all abuse types can lead to mental health disparities, sexual abuse and emotional abuse may have the most severe consequences for poorer mental health outcomes. This stands true in the gender diverse subgroup, with sexual and emotional abuse being most strongly associated with PTSD, GAD, cannabis dependence, self-harm, and attempted suicide. In this study, the prevalence of MDD and AUD was similar among individuals with diverse gender identities and cisgender individuals with experiences of child maltreatment.

Although we cannot ascertain causality from these survey data, the very high prevalence of child maltreatment among people with diverse gender identities may partially explain the higher prevalence of mental disorders and health risk behaviors in this group. These adversities exist within a wider cluster of difficulties in the lives of gender diverse people, including bullying, transphobia, stigma, and discrimination. As almost all the individuals with diverse gender identities in the ACMS had experienced child maltreatment, we were limited in our ability to disentangle the individual effects of child maltreatment and diverse gender identity on mental disorders and health risk behaviors. Nevertheless, the study results clearly show that mental disorders and health risk behaviors are common in people with diverse gender identities, most of whom have experienced child maltreatment, indicating that there are likely to be multiple factors needing to be addressed by clinicians supporting gender diverse people.

### Approaches to Prevention and Intervention

These findings have many implications, with two being particularly noteworthy. First, there is an urgent need to support youth and adults with diverse gender identities whose lives have been adversely impacted by the experience of child maltreatment and enable them to have access to mental health care. It is essential that trauma-informed approaches, including those attuned to the high likelihood of experiencing multiple types of child maltreatment be readily accessible and routinely offered to individuals with diverse gender identities presenting with any mental disorder and/or health risk behaviors (Goddard, 2021). Healthcare providers should consider the lived experience of people with diverse gender identities, including the experience of maltreatment and its influences on health and well-being. Comprehensive services should address the sequelae of historical child maltreatment in addition to contemporary gender-related distress and concurrent mental and physical health problems and social challenges (i.e., family conflict, stigma, discrimination). Healthcare providers—particularly those working with individuals with mental disorders and health risk behaviors—should also have suitable referral networks to provide appropriate care to those impacted by child maltreatment.

Second, there is an urgent need for social programs to prevent maltreatment of young people and adults with diverse gender identities. Further research is needed to investigate the efficacy of recommended population-based prevention strategies to protect people with diverse gender identities and reduce stigmatizing attitudes and behaviors among the wider community (MacMillan et al., 2009). Increased efforts are needed to better understand and empathically support people who identify different from their birth sex. The personal, familial, and social conflicts related to gender identity can be severe (Abreu et al., 2019; Benedini et al., 2016; Doidge et al., 2017) and, as this study suggests, may exacerbate the harm caused by child maltreatment. Greater acceptance of gender diversity in home, school, and community environments and in public discourse is essential to reduce interpersonal violence and foster improved wellbeing for individuals who identify as gender diverse (Roberts et al., 2013).

### Limitations

While the ACMS is nationally representative, individuals with diverse gender identities comprised a small proportion of the overall sample (*n* = 126, 1.5% unweighted). Accordingly, prevalence estimates and any observed associations with health and risk behavior were based on small numbers. It is possible that some important differences were unable to be ascertained due to limited statistical power. It is notable that there were very few gender diverse individuals who *did not* experience maltreatment (i.e., one in five reported no abuse or neglect) and therefore precluded analysis to identify whether maltreatment or identifying with a diverse gender was more strongly associated with worse outcomes. However, the fact that multiple significant differences were found between individuals with diverse gender identities and cisgender individuals for both the experience of child maltreatment and its association with mental disorders and health risk behaviors indicates the strength of these associations. A further limitation is that the ACMS did not collect information about the timing of the expression of diverse gender identities and the social and familial contexts that may have influenced gender expression. As such, we are unable to determine whether child maltreatment was experienced prior to or post expression of diverse gender identity. This limits our ability to determine potential drivers of the higher prevalence of child maltreatment among individuals with diverse gender identities.

## Conclusions

The prevalence of child maltreatment and the associated mental disorders is significantly more common in gender diverse individuals than in cisgender women and men, with ACMS data representative of this significant global issue. The experience of child maltreatment may contribute to the comparatively high levels of GAD, PTSD, self-harm, and suicide attempts among gender diverse individuals, although the strength of this conclusion is limited by the cross-sectional study design and the limited number of gender diverse individuals with *no* experience of maltreatment. It is clear that holistic mental health care is required for people with diverse gender identities. Consideration of the high likelihood of child maltreatment in people with diverse gender identities presenting for care, and providing appropriate, trauma-informed interventions is recommended. Population-wide global decreases in child maltreatment may have particularly strong benefits for this vulnerable group in society.

Moreover, creating a more supportive and accepting culture of gender diversity and provision of specific family and social supports may reduce the risk of child maltreatment among gender diverse youth. Governments across the developed world are actively debating care for transgender and gender diverse youth. Although our study presents data from Australia, there are strong reasons to believe that our results have value in other developed countries, with overall prevalence and patterns of child maltreatment and the associated mental conditions similar to other developed countries that have measured child maltreatment, including the United States, United Kingdom, Germany, Singapore, and the Netherlands (Stoltenborgh et al., 2011, 2012, 2013). While the debate about appropriate health care for young people with diverse gender identities is polarizing, the ACMS data make it clear that young people with high levels of distress, comorbid mental disorders, self-harming behaviors, and suicidal behaviors are likely to have experienced various forms of child abuse and neglect. Each of these factors alone would warrant targeted support and intervention. The high likelihood of multiple challenges in the lives of people with diverse gender identities underscores the need for multidisciplinary approaches to comprehensively address the health problems, adversity, and challenges that gender diverse youth face universally.

## Supplemental Material

sj-docx-1-jiv-10.1177_08862605241270077 – Supplemental material for Child Maltreatment, Mental Health Disorders, and Health Risk Behaviors in People With Diverse Gender Identities Supplemental material, sj-docx-1-jiv-10.1177_08862605241270077 for Child Maltreatment, Mental Health Disorders, and Health Risk Behaviors in People With Diverse Gender Identities by Monica Madzoska, David Lawrence, Daryl J. Higgins, Divna M. Haslam, Ben Mathews, Eva Malacova, Michael P. Dunne, Holly E. Erskine, Rosana Pacella, Franziska Meinck, Hannah J. Thomas and James G. Scott in Journal of Interpersonal Violence
