# Supplementary material for: Child Maltreatment, Mental Health Disorders, and Health Risk Behaviors in People With Diverse Gender Identities
Source: J Interpers Violence. 2024 Aug 17;40(9-10):2281–306. doi: 10.1177/08862605241270077 (PMC11951464; doi:10.1177/08862605241270077)
Supplement: sj-docx-1-jiv-10.1177_08862605241270077 – Supplemental material for Child Maltreatment, Mental Health Disorders, and Health Risk Behaviors in People With Diverse Gender Identities [file sj-docx-1-jiv-10.1177_08862605241270077.docx]

**Supplementary Tables**

**Supplementary Table 1: Prevalence of mental disorders by age group, gender identity, and whether experienced any child maltreatment**

| Age Group 16 - 24 years | | | | | | | |
| --- | --- | --- | --- | --- | --- | --- | --- |
|  | | Men | | Women | | Diverse genders | |
|  |  | % | 95% CI | % | 95% CI | % | 95% CI |
| Mental disorder | Experienced any child maltreatment | 1.0 | (0.3 - 1.7) | 1.9 | (0.7 - 3.0) | (a) |  |
| Posttraumatic Stress Disorder (Current) | No |  |  |  |  |  |  |
|  | Yes | 6.0 | (4.5 - 7.6) | 15.5 | (13.1 - 17.9) | 21.4 | (11.9 - 30.9) |
| Generalized Anxiety Disorder (Current) | No | 4.5 | (3.0 - 6.1) | 10.7 | (8.0 - 13.4) | (a) |  |
|  | Yes | 18.0 | (15.2 - 20.8) | 30.5 | (27.5 - 33.5) | 53.7 | (41.6 - 65.8) |
| Alcohol use disorder (Current) | No | 20.7 | (17.8 - 23.6) | 12.8 | (10.0 - 15.6) | (a) |  |
|  | Yes | 32.5 | (29.3 - 35.8) | 27.6 | (24.7 - 30.4) | 31.5 | (20.2 - 42.7) |
| Major Depressive Disorder (Lifetime) | No | 10.1 | (7.9 - 12.3) | 12.5 | (9.6 - 15.4) | (a) |  |
|  | Yes | 23.3 | (20.3 - 26.3) | 32.1 | (29.1 - 35.2) | 23.0 | (13.3 - 32.7) |
| Any Mental Disorder | No | 30.1 | (26.8 - 33.5) | 29.2 | (25.2 - 33.2) | 43.1 | (8.7 - 77.4) |
|  | Yes | 54.4 | (50.9 - 58.0) | 63.8 | (60.7 - 67.0) | 75.1 | (64.1 - 86.2) |
| Age Group 25 - 44 years | | | | | | | |
|  | | Men | | Women | | Diverse genders | |
|  |  | % | 95% CI | % | 95% CI | % | 95% CI |
| Mental disorder | Experienced any child maltreatment | 1.8 | (0.2 - 3.4) | (a) |  | (a) |  |
| Posttraumatic Stress Disorder (Current) | No |  |  |  |  |  |  |
|  | Yes | 7.5 | (5.2 - 9.8) | 9.3 | (6.9 - 11.7) | 28.8 | (7.7 - 49.9) |
| Generalized Anxiety Disorder (Current) | No | 5.9 | (3.2 - 8.6) | 7.2 | (3.9 - 10.5) | (a) |  |
|  | Yes | 17.7 | (14.3 - 21.1) | 20.4 | (17.1 - 23.8) | 53.9 | (30.1 - 77.7) |
| Alcohol use disorder (Current) | No | 22.9 | (18.2 - 27.6) | 10.4 | (6.7 - 14.1) | (a) |  |
|  | Yes | 35.9 | (31.7 - 40.2) | 23.4 | (19.9 - 27.0) | (a) |  |
| Major Depressive Disorder (Lifetime) | No | 8.3 | (5.3 - 11.2) | 12.6 | (8.7 - 16.5) | (a) |  |
|  | Yes | 25.1 | (21.3 - 28.9) | 31.3 | (27.5 - 35.1) | 20.2 | (2.6 - 37.8) |
| Any Mental Disorder | No | 29.2 | (24.2 - 34.2) | 23.5 | (18.4 - 28.7) | (a) |  |
|  | Yes | 57.0 | (52.7 - 61.4) | 55.4 | (51.3 - 59.5) | 58.9 | (35.4 - 82.5) |
| Age Group 45 years or more | | | | | | | |
|  | | Men | | Women | | Diverse genders | |
|  |  | % | 95% CI | % | 95% CI | % | 95% CI |
| Mental disorder | Experienced any child maltreatment | 1.1 | (0.2 - 2.0) | 1.2 | (0.2 - 2.2) | (a) |  |
| Posttraumatic Stress Disorder (Current) | No |  |  |  |  |  |  |
|  | Yes | 6.2 | (4.2 - 8.1) | 6.0 | (4.4 - 7.7) | (a) |  |
| Generalized Anxiety Disorder (Current) | No | 2.9 | (1.4 - 4.3) | 1.8 | (0.7 - 2.8) | (a) |  |
|  | Yes | 9.4 | (7.2 - 11.7) | 11.9 | (9.6 - 14.2) | (a) |  |
| Alcohol use disorder (Current) | No | 14.0 | (10.9 - 17.1) | 6.0 | (4.0 - 8.1) | (a) |  |
|  | Yes | 21.4 | (18.4 - 24.5) | 12.8 | (10.5 - 15.0) | (a) |  |
| Major Depressive Disorder (Lifetime) | No | 6.1 | (4.1 - 8.0) | 6.2 | (4.1 - 8.2) | (a) |  |
|  | Yes | 17.3 | (14.5 - 20.0) | 24.0 | (21.1 - 27.0) | (a) |  |
| Any Mental Disorder | No | 20.9 | (17.4 - 24.4) | 12.2 | (9.4 - 15.1) | (a) |  |
|  | Yes | 37.5 | (33.9 - 41.2) | 38.9 | (35.5 - 42.3) | (a) |  |

| All aged 16 years and over | | | | | | | |
| --- | --- | --- | --- | --- | --- | --- | --- |
|  | | Men | | Women | | Diverse genders | |
|  |  | % | 95% CI | % | 95% CI | % | 95% CI |
| Mental disorder | Experienced any child maltreatment | 1.3 | (0.6 - 2.0) | 1.2 | (0.5 - 1.9) | (a) |  |
| Posttraumatic Stress Disorder (Current) | No |  |  |  |  |  |  |
|  | Yes | 6.7 | (5.4 - 8.0) | 8.4 | (7.2 - 9.7) | 21.3 | (11.1 - 31.5) |
| Generalized Anxiety Disorder (Current) | No | 4.1 | (2.9 - 5.3) | 4.5 | (3.3 - 5.7) | (a) |  |
|  | Yes | 13.8 | (12.0 - 15.5) | 17.4 | (15.7 - 19.2) | 43.3 | (30.3 - 56.2) |
| Alcohol use disorder (Current) | No | 17.9 | (15.6 - 20.2) | 8.2 | (6.5 - 9.8) | (a) |  |
|  | Yes | 28.5 | (26.2 - 30.8) | 18.6 | (16.8 - 20.4) | 24.4 | (13.6 - 35.2) |
| Major Depressive Disorder (Lifetime) | No | 7.4 | (5.9 - 8.8) | 8.9 | (7.2 - 10.6) | (a) |  |
|  | Yes | 21.1 | (19.0 - 23.1) | 27.7 | (25.7 - 29.8) | 19.6 | (9.9 - 29.3) |
| Any Mental Disorder | No | 25.0 | (22.4 - 27.5) | 17.7 | (15.4 - 20.1) | 20.3 | (0.0 - 40.7) |
|  | Yes | 47.3 | (44.8 - 49.9) | 48.2 | (45.8 - 50.5) | 60.1 | (46.7 - 73.5) |

(95% CI) Lower and Upper Confidence Intervals

**Supplementary table 2: Prevalence of mental disorder, by gender identity and type of child maltreatment experienced**

|  | | Men | | Women | | Diverse genders | |
| --- | --- | --- | --- | --- | --- | --- | --- |
|  |  | % | 95% CI | % | 95% CI | % | 95% CI |
| Mental disorder | Type of child maltreatment | 1.3 | (0.6 - 2.0) | 1.2 | (0.5 - 1.9) | (a) |  |
| Posttraumatic Stress Disorder (Current) | No child maltreatment |  |  |  |  |  |  |
|  | Any child maltreatment | 6.7 | (5.4 - 8.0) | 8.4 | (7.2 - 9.7) | 21.3 | (11.1 - 31.5) |
|  | Emotional abuse | 10.5 | (8.1 - 12.9) | 12.1 | (10.1 - 14.0) | 28.0 | (14.6 - 41.3) |
|  | Neglect | 14.8 | (9.3 - 20.2) | 19.9 | (15.5 - 24.4) | 16.6 | (2.7 - 30.5) |
|  | Physical abuse | 9.7 | (7.6 - 11.8) | 11.0 | (9.0 - 12.9) | 25.6 | (11.2 - 39.9) |
|  | Sexual abuse | 9.6 | (7.0 - 12.2) | 11.1 | (9.2 - 13.0) | 24.1 | (11.5 - 36.6) |
|  | Exposure to domestic violence | 7.5 | (5.8 - 9.2) | 11.0 | (9.2 - 12.8) | 24.8 | (12.3 - 37.3) |
|  | One type of maltreatment | 2.8 | (1.4 - 4.2) | 2.5 | (1.2 - 3.8) | (a) |  |
|  | Two types of maltreatment | 4.9 | (2.7 - 7.0) | 7.2 | (4.6 - 9.9) | (a) |  |
|  | Three or more types of maltreatment | 13.4 | (10.2 - 16.5) | 13.9 | (11.6 - 16.3) | 28.2 | (14.3 - 42.1) |
| Generalized Anxiety Disorder (Current) | No child maltreatment | 4.1 | (2.9 - 5.3) | 4.5 | (3.3 - 5.7) | (a) |  |
|  | Any child maltreatment | 13.8 | (12.0 - 15.5) | 17.4 | (15.7 - 19.2) | 43.3 | (30.3 - 56.2) |
|  | Emotional abuse | 20.4 | (17.4 - 23.5) | 23.5 | (20.8 - 26.1) | 56.5 | (41.3 - 71.7) |
|  | Neglect | 24.0 | (17.8 - 30.2) | 30.8 | (25.3 - 36.2) | 58.2 | (34.2 - 82.2) |
|  | Physical abuse | 17.6 | (15.0 - 20.3) | 20.6 | (17.9 - 23.3) | 49.6 | (31.7 - 67.5) |
|  | Sexual abuse | 17.5 | (14.1 - 20.8) | 20.6 | (18.1 - 23.1) | 47.0 | (30.6 - 63.4) |
|  | Exposure to domestic violence | 15.7 | (13.4 - 18.0) | 20.1 | (17.8 - 22.4) | 49.7 | (34.2 - 65.2) |
|  | One type of maltreatment | 7.5 | (5.3 - 9.7) | 8.5 | (6.4 - 10.7) | 12.8 | (0.3 - 25.2) |
|  | Two types of maltreatment | 11.5 | (8.4 - 14.6) | 16.7 | (13.1 - 20.3) | 27.0 | (0.0 - 54.6) |
|  | Three or more types of maltreatment | 24.0 | (20.2 - 27.9) | 25.1 | (22.0 - 28.1) | 57.1 | (41.0 - 73.3) |
| Alcohol use disorder (Current) | No child maltreatment | 17.9 | (15.6 - 20.2) | 8.2 | (6.5 - 9.8) | (a) |  |
|  | Any child maltreatment | 28.5 | (26.2 - 30.8) | 18.6 | (16.8 - 20.4) | 24.4 | (13.6 - 35.2) |
|  | Emotional abuse | 28.0 | (24.7 - 31.4) | 21.5 | (18.9 - 24.0) | 27.8 | (15.6 - 40.0) |
|  | Neglect | 26.5 | (19.9 - 33.1) | 21.4 | (16.6 - 26.2) | 30.6 | (10.6 - 50.5) |
|  | Physical abuse | 29.1 | (26.0 - 32.2) | 20.0 | (17.3 - 22.7) | 26.3 | (11.3 - 41.3) |
|  | Sexual abuse | 30.7 | (26.6 - 34.8) | 22.5 | (19.9 - 25.0) | 36.2 | (20.6 - 51.7) |
|  | Exposure to domestic violence | 30.1 | (27.2 - 33.0) | 20.6 | (18.3 - 22.9) | 23.6 | (12.0 - 35.1) |
|  | One type of maltreatment | 27.7 | (24.1 - 31.3) | 12.3 | (9.7 - 14.8) | (a) |  |
|  | Two types of maltreatment | 27.3 | (23.0 - 31.6) | 17.7 | (14.2 - 21.2) | 36.3 | (1.9 - 70.7) |
|  | Three or more types of maltreatment | 30.7 | (26.7 - 34.8) | 24.2 | (21.2 - 27.2) | 27.2 | (14.1 - 40.2) |
| Major Depressive Disorder (Lifetime) | No child maltreatment | 7.4 | (5.9 - 8.8) | 8.9 | (7.2 - 10.6) | (a) |  |
|  | Any child maltreatment | 21.1 | (19.0 - 23.1) | 27.7 | (25.7 - 29.8) | 19.6 | (9.9 - 29.3) |
|  | Emotional abuse | 29.2 | (25.7 - 32.7) | 32.7 | (29.8 - 35.7) | 21.0 | (10.0 - 32.1) |
|  | Neglect | 31.3 | (24.3 - 38.2) | 31.2 | (25.6 - 36.7) | 18.2 | (2.0 - 34.4) |
|  | Physical abuse | 23.9 | (21.0 - 26.8) | 30.1 | (27.0 - 33.2) | 14.2 | (4.5 - 23.9) |
|  | Sexual abuse | 25.3 | (21.4 - 29.1) | 30.2 | (27.3 - 33.0) | 24.3 | (10.9 - 37.7) |
|  | Exposure to domestic violence | 23.3 | (20.7 - 25.9) | 30.5 | (27.8 - 33.2) | 17.9 | (7.5 - 28.3) |
|  | One type of maltreatment | 14.6 | (11.8 - 17.5) | 19.7 | (16.6 - 22.8) | (a) |  |
|  | Two types of maltreatment | 18.1 | (14.6 - 21.6) | 28.1 | (23.7 - 32.5) | (a) |  |
|  | Three or more types of maltreatment | 32.2 | (28.0 - 36.4) | 34.0 | (30.6 - 37.4) | 22.0 | (10.0 - 33.9) |
| Any Mental Disorder | No child maltreatment | 25.0 | (22.4 - 27.5) | 17.7 | (15.4 - 20.1) | 20.3 | (0.0 - 40.7) |
|  | Any child maltreatment | 47.3 | (44.8 - 49.9) | 48.2 | (45.8 - 50.5) | 60.1 | (46.7 - 73.5) |
|  | Emotional abuse | 55.1 | (51.3 - 59.0) | 57.9 | (54.8 - 61.1) | 69.1 | (53.9 - 84.3) |
|  | Neglect | 57.4 | (49.8 - 65.0) | 62.1 | (56.1 - 68.1) | 64.3 | (40.1 - 88.5) |
|  | Physical abuse | 50.6 | (47.1 - 54.0) | 53.8 | (50.3 - 57.2) | 63.6 | (45.6 - 81.6) |
|  | Sexual abuse | 51.8 | (47.3 - 56.3) | 53.9 | (50.8 - 57.1) | 71.2 | (55.9 - 86.6) |
|  | Exposure to domestic violence | 51.2 | (48.0 - 54.3) | 53.9 | (51.0 - 56.8) | 59.2 | (43.5 - 75.0) |
|  | One type of maltreatment | 40.1 | (36.2 - 44.1) | 32.3 | (28.6 - 36.0) | 32.5 | (4.5 - 60.6) |
|  | Two types of maltreatment | 43.1 | (38.3 - 47.9) | 45.8 | (41.0 - 50.6) | 61.5 | (26.7 - 96.2) |
|  | Three or more types of maltreatment | 60.5 | (56.1 - 64.9) | 62.4 | (58.9 - 66.0) | 68.0 | (51.7 - 84.2) |

**Supplementary figure 1: Prevalence of any mental disorder, by gender identity and experience of individual types of child maltreatment**

**
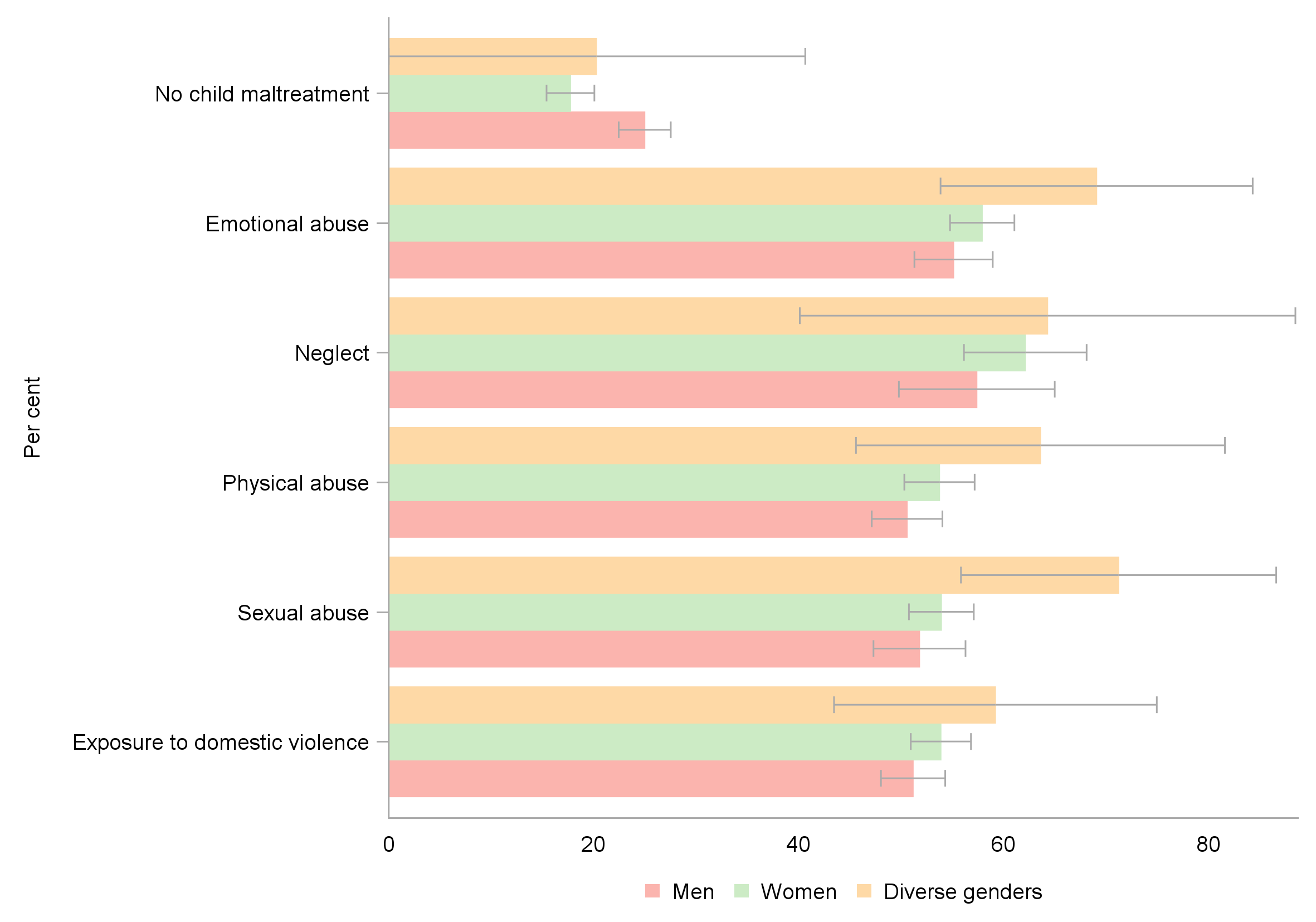
**

**Supplementary table 3: Prevalence of health risk behaviors by age group, gender identity, and whether experienced any child maltreatment**

| Age Group 16 - 24 years | | | | | | | |
| --- | --- | --- | --- | --- | --- | --- | --- |
|  | | Men | | Women | | Diverse genders | |
|  |  | % | 95% CI | % | 95% CI | % | 95% CI |
| Health risk behavior | Experienced any child maltreatment | 11.3 | (9.0 - 13.6) | 4.4 | (2.5 - 6.2) | (a) |  |
| Current smoker | No |  |  |  |  |  |  |
|  | Yes | 22.7 | (19.8 - 25.7) | 16.8 | (14.4 - 19.2) | 25.6 | (14.5 - 36.6) |
| Binge drinking | No | 10.5 | (8.3 - 12.7) | 4.0 | (2.4 - 5.5) | (a) |  |
|  | Yes | 11.4 | (9.3 - 13.5) | 6.8 | (5.3 - 8.4) | (a) |  |
| Cannabis dependence | No | 1.4 | (0.6 - 2.3) | (a) |  | (a) |  |
|  | Yes | 8.9 | (6.8 - 11.0) | 6.3 | (4.7 - 7.8) | 13.9 | (4.2 - 23.6) |
| Self-harm in past 12 months | No | 2.0 | (1.0 - 2.9) | 4.5 | (2.6 - 6.4) | (a) |  |
|  | Yes | 7.5 | (5.4 - 9.6) | 18.0 | (15.4 - 20.7) | 45.1 | (33.1 - 57.0) |
| Suicide attempt in past 12 months | No | 0.6 | (0.1 - 1.2) | (a) |  | (a) |  |
|  | Yes | 3.3 | (2.0 - 4.6) | 5.8 | (4.1 - 7.5) | 19.5 | (10.1 - 28.9) |
| Age Group 25 - 44 years | | | | | | | |
|  | | Men | | Women | | Diverse genders | |
|  |  | % | 95% CI | % | 95% CI | % | 95% CI |
| Health risk behavior | Experienced any child maltreatment | 19.3 | (14.7 - 24.0) | 10.1 | (6.3 - 13.9) | (a) |  |
| Current smoker | No |  |  |  |  |  |  |
|  | Yes | 28.2 | (24.1 - 32.2) | 22.2 | (18.6 - 25.8) | 31.5 | (8.3 - 54.7) |
| Binge drinking | No | 13.2 | (9.3 - 17.1) | 3.0 | (1.1 - 4.8) | (a) |  |
|  | Yes | 18.3 | (14.8 - 21.8) | 8.4 | (6.1 - 10.8) | (a) |  |
| Cannabis dependence | No | (a) |  | (a) |  | (a) |  |
|  | Yes | 6.2 | (4.0 - 8.4) | 3.3 | (1.7 - 4.9) | (a) |  |
| Self-harm in past 12 months | No | (a) |  | (a) |  | (a) |  |
|  | Yes | 5.0 | (3.0 - 6.9) | 5.6 | (3.8 - 7.5) | 27.8 | (6.4 - 49.2) |
| Suicide attempt in past 12 months | No | (a) |  | (a) |  | (a) |  |
|  | Yes | 1.6 | (0.6 - 2.7) | 1.8 | (0.8 - 2.9) | (a) |  |
| Age Group 45 years or more | | | | | | | |
|  | | Men | | Women | | Diverse genders | |
|  |  | % | 95% CI | % | 95% CI | % | 95% CI |
| Health risk behavior | Experienced any child maltreatment | 10.3 | (7.6 - 13.0) | 8.5 | (6.0 - 11.0) | (a) |  |
| Current smoker | No |  |  |  |  |  |  |
|  | Yes | 20.6 | (17.5 - 23.7) | 16.5 | (13.7 - 19.3) | (a) |  |
| Binge drinking | No | 13.2 | (10.2 - 16.2) | 3.8 | (1.9 - 5.6) | (a) |  |
|  | Yes | 18.7 | (15.7 - 21.7) | 8.7 | (6.7 - 10.7) | (a) |  |
| Cannabis dependence | No | (a) |  | (a) |  | (a) |  |
|  | Yes | 2.9 | (1.6 - 4.2) | 1.1 | (0.4 - 1.9) | (a) |  |
| Self-harm in past 12 months | No | (a) |  | (a) |  | (a) |  |
|  | Yes | 1.1 | (0.4 - 1.8) | 1.8 | (0.8 - 2.8) | (a) |  |
| Suicide attempt in past 12 months | No | (a) |  | (a) |  | (a) |  |
|  | Yes | 0.7 | (0.1 - 1.2) | (a) |  | (a) |  |

| All aged 16 years and over | | | | | | | |
| --- | --- | --- | --- | --- | --- | --- | --- |
|  | | Men | | Women | | Diverse genders | |
|  |  | % | 95% CI | % | 95% CI | % | 95% CI |
| Health risk behavior | Experienced any child maltreatment | 13.4 | (11.3 - 15.5) | 8.4 | (6.6 - 10.3) | (a) |  |
| Current smoker | No |  |  |  |  |  |  |
|  | Yes | 23.8 | (21.6 - 26.0) | 18.6 | (16.7 - 20.6) | 28.9 | (16.1 - 41.7) |
| Binge drinking | No | 12.8 | (10.8 - 14.9) | 3.6 | (2.4 - 4.8) | (a) |  |
|  | Yes | 17.6 | (15.6 - 19.6) | 8.4 | (7.0 - 9.7) | 14.2 | (4.2 - 24.2) |
| Cannabis dependence | No | 1.0 | (0.4 - 1.6) | (a) |  | (a) |  |
|  | Yes | 5.0 | (3.9 - 6.1) | 2.6 | (1.9 - 3.3) | 9.0 | (0.9 - 17.0) |
| Self-harm in past 12 months | No | 0.5 | (0.2 - 0.8) | 0.8 | (0.4 - 1.2) | (a) |  |
|  | Yes | 3.4 | (2.5 - 4.3) | 5.3 | (4.4 - 6.2) | 27.8 | (17.1 - 38.5) |
| Suicide attempt in past 12 months | No | 0.3 | (0.0 - 0.6) | (a) |  | (a) |  |
|  | Yes | 1.4 | (0.9 - 1.9) | 1.5 | (1.0 - 2.0) | 7.2 | (3.1 - 11.3) |

**Supplementary Table 4: Prevalence of health risk behaviors, by gender identity and type of child maltreatment experienced**

|  | | Men | | Women | | Diverse genders | |
| --- | --- | --- | --- | --- | --- | --- | --- |
|  |  | % | 95% CI | % | 95% CI | % | 95% CI |
| Health risk behavior | Type of child maltreatment | 13.4 | (11.3 - 15.5) | 8.4 | (6.6 - 10.3) | (a) |  |
| Current smoker | No child maltreatment |  |  |  |  |  |  |
|  | Any child maltreatment | 23.8 | (21.6 - 26.0) | 18.6 | (16.7 - 20.6) | 28.9 | (16.1 - 41.7) |
|  | Emotional abuse | 26.5 | (23.2 - 29.9) | 21.1 | (18.4 - 23.9) | 28.5 | (14.3 - 42.7) |
|  | Neglect | 30.2 | (23.1 - 37.2) | 23.0 | (17.7 - 28.4) | 39.5 | (15.1 - 63.8) |
|  | Physical abuse | 24.7 | (21.7 - 27.7) | 20.2 | (17.4 - 23.1) | 30.5 | (13.5 - 47.4) |
|  | Sexual abuse | 26.7 | (22.7 - 30.7) | 21.7 | (19.0 - 24.4) | 35.3 | (18.7 - 52.0) |
|  | Exposure to domestic violence | 25.8 | (23.1 - 28.6) | 18.2 | (15.8 - 20.6) | 33.7 | (18.5 - 48.9) |
|  | One type of maltreatment | 20.0 | (16.7 - 23.4) | 15.2 | (11.9 - 18.5) | (a) |  |
|  | Two types of maltreatment | 24.5 | (20.2 - 28.8) | 17.5 | (13.7 - 21.4) | (a) |  |
|  | Three or more types of maltreatment | 28.1 | (24.1 - 32.2) | 22.0 | (18.9 - 25.1) | 32.9 | (17.1 - 48.6) |
| Binge drinking | No child maltreatment | 12.8 | (10.8 - 14.9) | 3.6 | (2.4 - 4.8) | (a) |  |
|  | Any child maltreatment | 17.6 | (15.6 - 19.6) | 8.4 | (7.0 - 9.7) | 14.2 | (4.2 - 24.2) |
|  | Emotional abuse | 17.7 | (14.8 - 20.7) | 9.2 | (7.3 - 11.1) | 9.6 | (0.5 - 18.8) |
|  | Neglect | 16.7 | (10.9 - 22.6) | 6.8 | (4.0 - 9.6) | (a) |  |
|  | Physical abuse | 17.4 | (14.8 - 20.1) | 8.5 | (6.6 - 10.4) | 14.9 | (1.2 - 28.7) |
|  | Sexual abuse | 20.2 | (16.5 - 23.8) | 9.4 | (7.5 - 11.3) | 19.9 | (5.2 - 34.6) |
|  | Exposure to domestic violence | 18.0 | (15.5 - 20.4) | 8.1 | (6.4 - 9.7) | 12.1 | (2.3 - 22.0) |
|  | One type of maltreatment | 19.2 | (15.9 - 22.5) | 7.2 | (5.0 - 9.4) | (a) |  |
|  | Two types of maltreatment | 14.0 | (10.7 - 17.4) | 8.9 | (6.1 - 11.7) | (a) |  |
|  | Three or more types of maltreatment | 18.7 | (15.2 - 22.3) | 9.0 | (6.9 - 11.1) | 11.9 | (1.1 - 22.7) |
| Cannabis dependence | No child maltreatment | 1.0 | (0.4 - 1.6) | (a) |  | (a) |  |
|  | Any child maltreatment | 5.0 | (3.9 - 6.1) | 2.6 | (1.9 - 3.3) | 9.0 | (0.9 - 17.0) |
|  | Emotional abuse | 6.9 | (5.0 - 8.8) | 3.3 | (2.3 - 4.3) | 5.5 | (1.0 - 10.1) |
|  | Neglect | 7.6 | (3.8 - 11.4) | 3.9 | (2.0 - 5.8) | 9.0 | (0.0 - 18.2) |
|  | Physical abuse | 6.2 | (4.6 - 7.9) | 2.5 | (1.4 - 3.5) | 13.9 | (1.2 - 26.7) |
|  | Sexual abuse | 6.9 | (4.6 - 9.3) | 3.2 | (2.2 - 4.2) | 13.6 | (1.4 - 25.8) |
|  | Exposure to domestic violence | 5.4 | (4.1 - 6.8) | 3.0 | (2.0 - 4.0) | 6.7 | (1.2 - 12.3) |
|  | One type of maltreatment | 3.2 | (1.6 - 4.7) | 1.8 | (0.6 - 2.9) | (a) |  |
|  | Two types of maltreatment | 4.4 | (2.4 - 6.3) | 1.9 | (0.5 - 3.3) | (a) |  |
|  | Three or more types of maltreatment | 7.9 | (5.6 - 10.2) | 3.6 | (2.4 - 4.9) | 7.7 | (1.4 - 13.9) |
| Self-harm in past 12 months | No child maltreatment | 0.5 | (0.2 - 0.8) | 0.8 | (0.4 - 1.2) | (a) |  |
|  | Any child maltreatment | 3.4 | (2.5 - 4.3) | 5.3 | (4.4 - 6.2) | 27.8 | (17.1 - 38.5) |
|  | Emotional abuse | 4.4 | (2.9 - 5.8) | 7.6 | (6.2 - 9.1) | 36.3 | (22.4 - 50.2) |
|  | Neglect | 6.6 | (3.1 - 10.1) | 8.3 | (5.6 - 11.0) | 37.8 | (15.6 - 60.0) |
|  | Physical abuse | 3.5 | (2.4 - 4.6) | 5.7 | (4.4 - 7.0) | 32.4 | (17.3 - 47.6) |
|  | Sexual abuse | 5.3 | (3.2 - 7.3) | 6.9 | (5.5 - 8.3) | 38.6 | (23.2 - 53.9) |
|  | Exposure to domestic violence | 3.9 | (2.7 - 5.1) | 6.3 | (5.0 - 7.5) | 29.4 | (16.6 - 42.2) |
|  | One type of maltreatment | 2.2 | (1.0 - 3.3) | 1.9 | (1.0 - 2.8) | (a) |  |
|  | Two types of maltreatment | 3.8 | (1.9 - 5.8) | 5.2 | (3.2 - 7.3) | (a) |  |
|  | Three or more types of maltreatment | 4.7 | (3.0 - 6.4) | 8.1 | (6.4 - 9.8) | 39.2 | (24.0 - 54.5) |
| Suicide attempt in past 12 months | No child maltreatment | 0.3 | (0.0 - 0.6) | (a) |  | (a) |  |
|  | Any child maltreatment | 1.4 | (0.9 - 1.9) | 1.5 | (1.0 - 2.0) | 7.2 | (3.1 - 11.3) |
|  | Emotional abuse | 2.1 | (1.1 - 3.0) | 2.3 | (1.5 - 3.1) | 9.4 | (3.9 - 15.0) |
|  | Neglect | 4.2 | (1.2 - 7.3) | 2.7 | (1.2 - 4.3) | 10.7 | (1.9 - 19.6) |
|  | Physical abuse | 2.0 | (1.2 - 2.9) | 1.7 | (1.1 - 2.4) | 7.6 | (2.2 - 13.1) |
|  | Sexual abuse | 2.2 | (0.9 - 3.4) | 2.0 | (1.3 - 2.7) | 10.0 | (3.8 - 16.1) |
|  | Exposure to domestic violence | 1.6 | (0.9 - 2.4) | 1.9 | (1.2 - 2.5) | 8.6 | (3.3 - 13.8) |
|  | One type of maltreatment | 0.6 | (0.0 - 1.1) | 0.4 | (0.0 - 0.8) | (a) |  |
|  | Two types of maltreatment | 1.1 | (0.2 - 2.0) | 1.0 | (0.3 - 1.7) | (a) |  |
|  | Three or more types of maltreatment | 2.7 | (1.4 - 4.1) | 2.7 | (1.7 - 3.7) | 9.8 | (3.8 - 15.7) |

**Supplementary table 5: Likelihood of post-traumatic stress disorder, by gender identity and experience of child maltreatment**

|  |  | |  | |
| --- | --- | --- | --- | --- |
| Parameter | Unadjusted (a) | | Adjusted (b) | |
|  | OR | 95% CI | OR | 95% CI |
| Gender and experience of child maltreatment— |  |  |  |  |
| Men No Maltreatment | 1 | (Ref) | 1 | (Ref) |
| Women Any Maltreatment | 7.0 | (4.0 - 12.3) | 4.7 | (2.7 - 8.5) |
| Women No Maltreatment | 0.9 | (0.4 - 2.1) | 0.9 | (0.4 - 1.9) |
| Men Any Maltreatment | 5.4 | (3.0 - 9.6) | 4.1 | (2.3 - 7.4) |
| Diverse genders Any Maltreatment | 17.6 | (7.8 - 39.6) | 9.5 | (3.7 - 24.2) |
| Age group— |  |  |  |  |
| 16 - 24 years | 1 | (Ref) | 1 | (Ref) |
| 25 - 44 years | 0.8 | (0.6 - 1.0) | 0.7 | (0.5 - 0.9) |
| 45 years or more | 0.5 | (0.4 - 0.7) | 0.6 | (0.4 - 0.7) |
| SEIFA index of relative socio-economic disadvantage— |  |  |  |  |
| Highest quintile |  |  | 1 | (Ref) |
| 2nd quintile |  |  | 1.1 | (0.7 - 1.6) |
| 3rd quintile |  |  | 1.4 | (1.0 - 2.0) |
| 4th quintile |  |  | 1.3 | (0.9 - 1.9) |
| Lowest quintile |  |  | 1.4 | (0.9 - 2.1) |
| Financial hardship in past 12 months— |  |  |  |  |
| No |  |  | 1 | (Ref) |
| Don't know |  |  | 0.9 | (0.2 - 3.1) |
| Yes |  |  | 3.3 | (2.5 - 4.5) |
| Financial hardship in childhood— |  |  |  |  |
| Never |  |  | 1 | (Ref) |
| Don't know |  |  | 0.7 | (0.1 - 3.8) |
| Not very often |  |  | 1.4 | (1.0 - 1.9) |
| Somewhat often |  |  | 1.6 | (1.1 - 2.3) |
| Very often |  |  | 2.3 | (1.6 - 3.4) |

(a) Adjusted for age group

(b) Adjusted for age group, social economic indexes for area (SEIFA), financial hardship in past 12 months, and financial hardship in childhood

**Supplementary table 6: Likelihood of generalized anxiety disorder, by gender identity and experience of child maltreatment**

|  |  | |  | |
| --- | --- | --- | --- | --- |
| Parameter | Unadjusted (a) | | Adjusted (b) | |
|  | OR | 95% CI | OR | 95% CI |
| Gender and experience of child maltreatment— |  |  |  |  |
| Men No Maltreatment | 1 | (Ref) | 1 | (Ref) |
| Women Any Maltreatment | 5.0 | (3.6 - 6.9) | 3.8 | (2.7 - 5.3) |
| Women No Maltreatment | 1.2 | (0.8 - 1.7) | 1.1 | (0.7 - 1.7) |
| Men Any Maltreatment | 3.7 | (2.6 - 5.2) | 3.0 | (2.2 - 4.3) |
| Diverse genders Any Maltreatment | 14.4 | (8.0 - 25.8) | 8.6 | (4.6 - 16.2) |
| Age group— |  |  |  |  |
| 16 - 24 years | 1 | (Ref) | 1 | (Ref) |
| 25 - 44 years | 0.7 | (0.6 - 0.9) | 0.7 | (0.6 - 0.8) |
| 45 years or more | 0.4 | (0.3 - 0.4) | 0.4 | (0.3 - 0.4) |
| SEIFA index of relative socio-economic disadvantage— |  |  |  |  |
| Highest quintile |  |  | 1 | (Ref) |
| 2nd quintile |  |  | 0.8 | (0.6 - 1.0) |
| 3rd quintile |  |  | 0.9 | (0.7 - 1.1) |
| 4th quintile |  |  | 1.0 | (0.8 - 1.3) |
| Lowest quintile |  |  | 1.0 | (0.7 - 1.3) |
| Financial hardship in past 12 months— |  |  |  |  |
| No |  |  | 1 | (Ref) |
| Don't know |  |  | 1.9 | (0.9 - 4.1) |
| Yes |  |  | 2.5 | (2.0 - 3.2) |
| Financial hardship in childhood— |  |  |  |  |
| Never |  |  | 1 | (Ref) |
| Don't know |  |  | 0.8 | (0.3 - 2.1) |
| Not very often |  |  | 1.3 | (1.1 - 1.7) |
| Somewhat often |  |  | 1.5 | (1.2 - 2.0) |
| Very often |  |  | 2.1 | (1.6 - 2.9) |

(a) Adjusted for age group

(b) Adjusted for age group, SEIFA, financial hardship in past 12 months, and financial hardship in childhood

**Supplementary table 7: Likelihood of major depressive disorder, by gender identity and experience of child maltreatment**

|  |  | |  | |
| --- | --- | --- | --- | --- |
| Parameter | Unadjusted (a) | | Adjusted (b) | |
|  | OR | 95% CI | OR | 95% CI |
| Gender and experience of child maltreatment— |  |  |  |  |
| Men No Maltreatment | 1 | (Ref) | 1 | (Ref) |
| Women Any Maltreatment | 4.8 | (3.8 - 6.1) | 4.4 | (3.4 - 5.6) |
| Women No Maltreatment | 1.3 | (0.9 - 1.7) | 1.3 | (0.9 - 1.7) |
| Men Any Maltreatment | 3.3 | (2.6 - 4.2) | 3.0 | (2.4 - 3.9) |
| Diverse genders Any Maltreatment | 2.7 | (1.4 - 5.2) | 2.5 | (1.3 - 4.8) |
| Age group— |  |  |  |  |
| 16 - 24 years | 1 | (Ref) | 1 | (Ref) |
| 25 - 44 years | 1.0 | (0.8 - 1.1) | 1.0 | (0.8 - 1.1) |
| 45 years or more | 0.6 | (0.5 - 0.7) | 0.6 | (0.6 - 0.7) |
| SEIFA index of relative socio-economic disadvantage— |  |  |  |  |
| Highest quintile |  |  | 1 | (Ref) |
| 2nd quintile |  |  | 0.9 | (0.7 - 1.1) |
| 3rd quintile |  |  | 1.1 | (0.9 - 1.3) |
| 4th quintile |  |  | 1.0 | (0.8 - 1.3) |
| Lowest quintile |  |  | 0.8 | (0.6 - 1.0) |
| Financial hardship in past 12 months— |  |  |  |  |
| No |  |  | 1 | (Ref) |
| Don't know |  |  | 0.4 | (0.1 - 0.9) |
| Yes |  |  | 1.1 | (0.9 - 1.4) |
| Financial hardship in childhood— |  |  |  |  |
| Never |  |  | 1 | (Ref) |
| Don't know |  |  | 1.3 | (0.6 - 2.7) |
| Not very often |  |  | 1.4 | (1.2 - 1.6) |
| Somewhat often |  |  | 1.3 | (1.1 - 1.7) |
| Very often |  |  | 1.4 | (1.1 - 1.9) |

(a) Adjusted for age group

(b) Adjusted for age group, SEIFA, financial hardship in past 12 months, and financial hardship in childhood

**Supplementary table 8: Likelihood of alcohol use disorder, by gender identity and experience of child maltreatment**

|  |  | |  | |
| --- | --- | --- | --- | --- |
| Parameter | Unadjusted (a) | | Adjusted (b) | |
|  | OR | 95% CI | OR | 95% CI |
| Gender and experience of child maltreatment— |  |  |  |  |
| Men No Maltreatment | 1 | (Ref) | 1 | (Ref) |
| Women Any Maltreatment | 1.0 | (0.8 - 1.3) | 0.9 | (0.7 - 1.1) |
| Women No Maltreatment | 0.4 | (0.3 - 0.6) | 0.4 | (0.3 - 0.5) |
| Men Any Maltreatment | 1.8 | (1.5 - 2.2) | 1.7 | (1.4 - 2.0) |
| Diverse genders Any Maltreatment | 1.2 | (0.7 - 2.3) | 1.0 | (0.5 - 1.9) |
| Age group— |  |  |  |  |
| 16 - 24 years | 1 | (Ref) | 1 | (Ref) |
| 25 - 44 years | 1.0 | (0.8 - 1.1) | 0.9 | (0.8 - 1.1) |
| 45 years or more | 0.5 | (0.4 - 0.6) | 0.5 | (0.4 - 0.6) |
| SEIFA index of relative socio-economic disadvantage— |  |  |  |  |
| Highest quintile |  |  | 1 | (Ref) |
| 2nd quintile |  |  | 0.8 | (0.6 - 0.9) |
| 3rd quintile |  |  | 0.8 | (0.6 - 0.9) |
| 4th quintile |  |  | 0.8 | (0.6 - 1.0) |
| Lowest quintile |  |  | 0.6 | (0.5 - 0.8) |
| Financial hardship in past 12 months— |  |  |  |  |
| No |  |  | 1 | (Ref) |
| Don't know |  |  | 0.4 | (0.1 - 1.0) |
| Yes |  |  | 1.6 | (1.3 - 2.0) |
| Financial hardship in childhood— |  |  |  |  |
| Never |  |  | 1 | (Ref) |
| Don't know |  |  | 0.8 | (0.4 - 1.8) |
| Not very often |  |  | 1.2 | (1.0 - 1.4) |
| Somewhat often |  |  | 1.3 | (1.1 - 1.7) |
| Very often |  |  | 1.3 | (1.0 - 1.7) |

(a) Adjusted for age group

(b) Adjusted for age group, SEIFA, financial hardship in past 12 months, and financial hardship in childhood

**Supplementary table 9: Likelihood of any mental disorder, by gender identity and experience of child maltreatment**

|  |  | |  | |
| --- | --- | --- | --- | --- |
| Parameter | Unadjusted (a) | | Adjusted (b) | |
|  | OR | 95% CI | OR | 95% CI |
| Gender and experience of child maltreatment— |  |  |  |  |
| Men No Maltreatment | 1 | (Ref) | 1 | (Ref) |
| Women Any Maltreatment | 2.8 | (2.4 - 3.4) | 2.4 | (2.0 - 2.8) |
| Women No Maltreatment | 0.7 | (0.5 - 0.8) | 0.7 | (0.5 - 0.8) |
| Men Any Maltreatment | 2.7 | (2.3 - 3.2) | 2.4 | (2.0 - 2.8) |
| Diverse genders Any Maltreatment | 3.7 | (2.1 - 6.6) | 2.8 | (1.6 - 5.1) |
| Age group— |  |  |  |  |
| 16 - 24 years | 1 | (Ref) | 1 | (Ref) |
| 25 - 44 years | 0.9 | (0.8 - 1.0) | 0.8 | (0.7 - 0.9) |
| 45 years or more | 0.4 | (0.4 - 0.5) | 0.4 | (0.4 - 0.5) |
| SEIFA index of relative socio-economic disadvantage— |  |  |  |  |
| Highest quintile |  |  | 1 | (Ref) |
| 2nd quintile |  |  | 0.8 | (0.7 - 0.9) |
| 3rd quintile |  |  | 0.9 | (0.8 - 1.1) |
| 4th quintile |  |  | 0.9 | (0.7 - 1.0) |
| Lowest quintile |  |  | 0.7 | (0.6 - 0.9) |
| Financial hardship in past 12 months— |  |  |  |  |
| No |  |  | 1 | (Ref) |
| Don't know |  |  | 0.6 | (0.4 - 1.2) |
| Yes |  |  | 1.9 | (1.6 - 2.3) |
| Financial hardship in childhood— |  |  |  |  |
| Never |  |  | 1 | (Ref) |
| Don't know |  |  | 1.0 | (0.5 - 1.9) |
| Not very often |  |  | 1.3 | (1.2 - 1.5) |
| Somewhat often |  |  | 1.5 | (1.3 - 1.9) |
| Very often |  |  | 1.8 | (1.4 - 2.3) |

(a) Adjusted for age group

(b) Adjusted for age group, SEIFA, financial hardship in past 12 months, and financial hardship in childhood

**Supplementary table 10: Likelihood of current smoking, by gender identity and experience of child maltreatment**

|  |  | |  | |
| --- | --- | --- | --- | --- |
| Parameter | Unadjusted (a) | | Adjusted (b) | |
|  | OR | 95% CI | OR | 95% CI |
| Gender and experience of child maltreatment— |  |  |  |  |
| Men No Maltreatment | 1 | (Ref) | 1 | (Ref) |
| Women Any Maltreatment | 1.5 | (1.2 - 1.8) | 1.2 | (1.0 - 1.5) |
| Women No Maltreatment | 0.6 | (0.4 - 0.8) | 0.6 | (0.4 - 0.8) |
| Men Any Maltreatment | 2.0 | (1.6 - 2.5) | 1.8 | (1.4 - 2.2) |
| Diverse genders Any Maltreatment | 2.6 | (1.3 - 4.9) | 1.7 | (0.9 - 3.2) |
| Age group— |  |  |  |  |
| 16 - 24 years | 1 | (Ref) | 1 | (Ref) |
| 25 - 44 years | 1.5 | (1.3 - 1.8) | 1.5 | (1.2 - 1.7) |
| 45 years or more | 1.0 | (0.8 - 1.2) | 1.0 | (0.9 - 1.2) |
| SEIFA index of relative socio-economic disadvantage— |  |  |  |  |
| Highest quintile |  |  | 1 | (Ref) |
| 2nd quintile |  |  | 1.5 | (1.2 - 1.9) |
| 3rd quintile |  |  | 1.1 | (0.9 - 1.4) |
| 4th quintile |  |  | 0.9 | (0.7 - 1.2) |
| Lowest quintile |  |  | 1.5 | (1.1 - 1.9) |
| Financial hardship in past 12 months— |  |  |  |  |
| No |  |  | 1 | (Ref) |
| Don't know |  |  | 1.7 | (0.8 - 3.9) |
| Yes |  |  | 3.0 | (2.4 - 3.6) |
| Financial hardship in childhood— |  |  |  |  |
| Never |  |  | 1 | (Ref) |
| Don't know |  |  | 0.4 | (0.1 - 1.1) |
| Not very often |  |  | 0.9 | (0.8 - 1.1) |
| Somewhat often |  |  | 1.2 | (1.0 - 1.6) |
| Very often |  |  | 1.2 | (0.9 - 1.5) |

(a) Adjusted for age group

(b) Adjusted for age group, SEIFA, financial hardship in past 12 months, and financial hardship in childhood

**Supplementary table 11: Likelihood of binge drinking, by gender identity and experience of child maltreatment**

|  |  | |  | |
| --- | --- | --- | --- | --- |
| Parameter | Unadjusted (a) | | Adjusted (b) | |
|  | OR | 95% CI | OR | 95% CI |
| Gender and experience of child maltreatment— |  |  |  |  |
| Men No Maltreatment | 1 | (Ref) | 1 | (Ref) |
| Women Any Maltreatment | 0.6 | (0.5 - 0.8) | 0.5 | (0.4 - 0.7) |
| Women No Maltreatment | 0.2 | (0.2 - 0.4) | 0.2 | (0.2 - 0.4) |
| Men Any Maltreatment | 1.4 | (1.2 - 1.8) | 1.3 | (1.0 - 1.6) |
| Diverse genders Any Maltreatment | 1.2 | (0.5 - 2.9) | 1.0 | (0.4 - 2.4) |
| Age group— |  |  |  |  |
| 16 - 24 years | 1 | (Ref) | 1 | (Ref) |
| 25 - 44 years | 1.4 | (1.2 - 1.7) | 1.4 | (1.1 - 1.7) |
| 45 years or more | 1.5 | (1.2 - 1.8) | 1.5 | (1.2 - 1.8) |
| SEIFA index of relative socio-economic disadvantage— |  |  |  |  |
| Highest quintile |  |  | 1 | (Ref) |
| 2nd quintile |  |  | 1.2 | (0.9 - 1.5) |
| 3rd quintile |  |  | 1.0 | (0.7 - 1.3) |
| 4th quintile |  |  | 0.9 | (0.7 - 1.2) |
| Lowest quintile |  |  | 1.0 | (0.7 - 1.3) |
| Financial hardship in past 12 months— |  |  |  |  |
| No |  |  | 1 | (Ref) |
| Don't know |  |  | 0.4 | (0.1 - 1.6) |
| Yes |  |  | 1.7 | (1.3 - 2.2) |
| Financial hardship in childhood— |  |  |  |  |
| Never |  |  | 1 | (Ref) |
| Don't know |  |  | 1.2 | (0.5 - 2.8) |
| Not very often |  |  | 1.3 | (1.0 - 1.6) |
| Somewhat often |  |  | 1.2 | (0.9 - 1.6) |
| Very often |  |  | 1.5 | (1.1 - 2.1) |

(a) Adjusted for age group

(b) Adjusted for age group, SEIFA, financial hardship in past 12 months, and financial hardship in childhood

**Supplementary table 12: Likelihood of cannabis dependence, by gender identity and experience of child maltreatment**

|  |  | |  | |
| --- | --- | --- | --- | --- |
| Parameter | Unadjusted (a) | | Adjusted (b) | |
|  | OR | 95% CI | OR | 95% CI |
| Gender and experience of child maltreatment— |  |  |  |  |
| Men No Maltreatment | 1 | (Ref) | 1 | (Ref) |
| Women Any Maltreatment | 2.7 | (1.4 - 5.6) | 2.1 | (1.0 - 4.4) |
| Women No Maltreatment | 0.1 | (0.0 - 0.4) | 0.1 | (0.0 - 0.4) |
| Men Any Maltreatment | 5.4 | (2.7 - 10.6) | 4.5 | (2.3 - 9.1) |
| Diverse genders Any Maltreatment | 7.4 | (2.2 - 25.2) | 4.3 | (1.2 - 15.4) |
| Age group— |  |  |  |  |
| 16 - 24 years | 1 | (Ref) | 1 | (Ref) |
| 25 - 44 years | 0.6 | (0.4 - 0.8) | 0.6 | (0.4 - 0.8) |
| 45 years or more | 0.3 | (0.2 - 0.4) | 0.3 | (0.2 - 0.4) |
| SEIFA index of relative socio-economic disadvantage— |  |  |  |  |
| Highest quintile |  |  | 1 | (Ref) |
| 2nd quintile |  |  | 1.0 | (0.6 - 1.7) |
| 3rd quintile |  |  | 0.9 | (0.5 - 1.5) |
| 4th quintile |  |  | 0.8 | (0.5 - 1.4) |
| Lowest quintile |  |  | 1.1 | (0.6 - 2.0) |
| Financial hardship in past 12 months— |  |  |  |  |
| No |  |  | 1 | (Ref) |
| Don't know |  |  | 2.2 | (0.5 - 9.2) |
| Yes |  |  | 3.8 | (2.5 - 5.7) |
| Financial hardship in childhood— |  |  |  |  |
| Never |  |  | 1 | (Ref) |
| Don't know |  |  | 1.6 | (0.4 - 6.2) |
| Not very often |  |  | 1.4 | (0.9 - 2.1) |
| Somewhat often |  |  | 1.1 | (0.6 - 1.9) |
| Very often |  |  | 1.1 | (0.6 - 2.0) |

(a) Adjusted for age group

(b) Adjusted for age group, SEIFA, financial hardship in past 12 months, and financial hardship in childhood

**Supplementary table 13: Likelihood of self-harm in the past 12 months, by gender identity and experience of child maltreatment**

|  |  | |  | |
| --- | --- | --- | --- | --- |
| Parameter | Unadjusted (a) | | Adjusted (b) | |
|  | OR | 95% CI | OR | 95% CI |
| Gender and experience of child maltreatment— |  |  |  |  |
| Men No Maltreatment | 1 | (Ref) | 1 | (Ref) |
| Women Any Maltreatment | 12.0 | (6.0 - 23.8) | 10.0 | (5.0 - 20.0) |
| Women No Maltreatment | 1.8 | (0.8 - 4.1) | 1.7 | (0.8 - 3.9) |
| Men Any Maltreatment | 7.3 | (3.6 - 14.8) | 6.4 | (3.2 - 13.1) |
| Diverse genders Any Maltreatment | 52.4 | (22.0 - 123.0) | 40.7 | (16.6 - 99.6) |
| Age group— |  |  |  |  |
| 16 - 24 years | 1 | (Ref) | 1 | (Ref) |
| 25 - 44 years | 0.3 | (0.3 - 0.5) | 0.3 | (0.2 - 0.4) |
| 45 years or more | 0.1 | (0.1 - 0.1) | 0.1 | (0.1 - 0.1) |
| SEIFA index of relative socio-economic disadvantage— |  |  |  |  |
| Highest quintile |  |  | 1 | (Ref) |
| 2nd quintile |  |  | 0.9 | (0.6 - 1.4) |
| 3rd quintile |  |  | 0.9 | (0.6 - 1.3) |
| 4th quintile |  |  | 0.9 | (0.6 - 1.4) |
| Lowest quintile |  |  | 1.2 | (0.7 - 1.9) |
| Financial hardship in past 12 months— |  |  |  |  |
| No |  |  | 1 | (Ref) |
| Don't know |  |  | 1.0 | (0.3 - 3.5) |
| Yes |  |  | 2.0 | (1.4 - 2.8) |
| Financial hardship in childhood— |  |  |  |  |
| Never |  |  | 1 | (Ref) |
| Don't know |  |  | 1.8 | (0.4 - 8.0) |
| Not very often |  |  | 1.3 | (0.9 - 1.8) |
| Somewhat often |  |  | 1.6 | (1.1 - 2.5) |
| Very often |  |  | 1.0 | (0.6 - 1.6) |

(a) Adjusted for age group

(b) Adjusted for age group, SEIFA, financial hardship in past 12 months, and financial hardship in childhood

**Supplementary table 14: Likelihood of suicide attempt in the past 12 months, by gender identity and experience of child maltreatment**

|  |  | |  | |
| --- | --- | --- | --- | --- |
| Parameter | Unadjusted (a) | | Adjusted (b) | |
|  | OR | 95% CI | OR | 95% CI |
| Gender and experience of child maltreatment— |  |  |  |  |
| Men No Maltreatment | 1 | (Ref) | 1 | (Ref) |
| Women Any Maltreatment | 5.3 | (1.9 - 15.4) | 3.8 | (1.3 - 11.0) |
| Women No Maltreatment | 1.0 | (0.2 - 5.4) | 0.9 | (0.2 - 4.9) |
| Men Any Maltreatment | 4.8 | (1.6 - 14.0) | 3.9 | (1.3 - 11.5) |
| Diverse genders Any Maltreatment | 16.0 | (5.2 - 49.1) | 8.7 | (2.7 - 27.4) |
| Age group— |  |  |  |  |
| 16 - 24 years | 1 | (Ref) | 1 | (Ref) |
| 25 - 44 years | 0.3 | (0.2 - 0.5) | 0.3 | (0.2 - 0.5) |
| 45 years or more | 0.1 | (0.1 - 0.2) | 0.1 | (0.1 - 0.2) |
| SEIFA index of relative socio-economic disadvantage— |  |  |  |  |
| Highest quintile |  |  | 1 | (Ref) |
| 2nd quintile |  |  | 2.0 | (1.0 - 4.1) |
| 3rd quintile |  |  | 1.6 | (0.8 - 3.4) |
| 4th quintile |  |  | 1.6 | (0.7 - 3.6) |
| Lowest quintile |  |  | 2.6 | (1.2 - 5.6) |
| Financial hardship in past 12 months— |  |  |  |  |
| No |  |  | 1 | (Ref) |
| Don't know |  |  | 3.6 | (0.9 - 14.5) |
| Yes |  |  | 3.5 | (2.0 - 6.0) |
| Financial hardship in childhood— |  |  |  |  |
| Never |  |  | 1 | (Ref) |
| Don't know |  |  | 0.0 | (0.0 - 0.0) |
| Not very often |  |  | 1.1 | (0.6 - 1.9) |
| Somewhat often |  |  | 1.6 | (0.8 - 3.1) |
| Very often |  |  | 1.0 | (0.4 - 2.0) |

(a) Adjusted for age group

(b) Adjusted for age group, SEIFA, financial hardship in past 12 months, and financial hardship in childhood
